# Comparative genome sequencing reveals insights into the dynamics of *Wolbachia* in native and invasive cherry fruit flies

**DOI:** 10.1111/mec.15923

**Published:** 2021-05-07

**Authors:** Thomas M. Wolfe, Daniel J. Bruzzese, Lisa Klasson, Erika Corretto, Sonja Lečić, Christian Stauffer, Jeffrey L. Feder, Hannes Schuler

**Affiliations:** ^1^ Department of Forest and Soil Sciences Boku, University of Natural Resources and Life Sciences Vienna Austria; ^2^ Department of Biological Sciences University of Notre Dame Notre Dame Indiana USA; ^3^ Molecular Evolution Department of Cell and Molecular Biology Science for Life Laboratory Uppsala University Uppsala Sweden; ^4^ Faculty of Science and Technology Free University of Bozen‐Bolzano Bozen‐Bolzano Italy; ^5^ Department of Evolutionary Biology Ludwig‐Maximilians University Munich Germany; ^6^ Competence Centre for Plant Health Free University of Bozen‐Bolzano Bozen‐Bolzano Italy

**Keywords:** horizontal transfer, invasive species, prophage, *Rhagoletis cerasi*, *Rhagoletis cingulata*, *Wolbachia*

## Abstract

*Wolbachia* is a maternally inherited obligate endosymbiont that can induce a wide spectrum of effects in its host, ranging from mutualism to reproductive parasitism. At the genomic level, recombination within and between strains, transposable elements, and horizontal transfer of strains between host species make *Wolbachia* an evolutionarily dynamic bacterial system. The invasive cherry fruit fly *Rhagoletis cingulata* arrived in Europe from North America ~40 years ago, where it now co‐occurs with the native cherry pest *R*. *cerasi*. This shared distribution has been proposed to have led to the horizontal transfer of different *Wolbachia* strains between the two species. To better understand transmission dynamics, we performed a comparative genome study of the strain *w*Cin2 in its native United States and invasive European populations of *R*. *cingulata* with *w*Cer2 in European *R*. *cerasi*. Previous multilocus sequence genotyping (MLST) of six genes implied that the source of *w*Cer2 in *R*. *cerasi* was *w*Cin2 from *R*. *cingulata*. However, we report genomic evidence discounting the recent horizontal transfer hypothesis for the origin of *w*Cer2. Despite near identical sequences for the MLST markers, substantial sequence differences for other loci were found between *w*Cer2 and *w*Cin2, as well as structural rearrangements, and differences in prophage, repetitive element, gene content, and cytoplasmic incompatibility inducing genes. Our study highlights the need for whole‐genome sequencing rather than relying on MLST markers for resolving *Wolbachia* strains and assessing their evolutionary dynamics.

## 1 INTRODUCTION

*Wolbachia* is a highly successful endosymbiont that can manipulate host reproduction to ensure efficient vertical transmission from mother to offspring (Werren et al., 2008). Cytoplasmic incompatibility (CI) is the most common form of reproductive manipulation, where crosses between uninfected females and infected males result in embryonic death of offspring. However, if an infected male mates with a female infected with a compatible strain, offspring are rescued from death (Engelstädter & Hurst, 2009; Shropshire et al., 2020; Turelli & Hoffman, 1991; Yen & Barr, 1971). This strategy gives infected females a fitness advantage in a population over uninfected females and has contributed to *Wolbachia* infecting more than 50% of all terrestrial arthropods (Weinert et al., 2015). *Wolbachia* may also provide fitness advantages to the hosts by conferring protection against viruses or other microbes (Chrostek et al., 2013; Hedges et al., 2008; Teixeira et al., 2008), increasing fecundity (Fast et al., 2011; Fry et al., 2004; Moriyama et al., 2015), or affecting survivorship and longevity (Maistrenko et al., 2016). Together, these strategies serve to increase *Wolbachia*'s fitness and aid its spread to high frequencies within host species (Baković et al., 2018; Kriesner et al., 2016; Turelli et al., 2018; Turelli & Hoffmann, 1991).

Although vertical transmission is considered the primary mode of *Wolbachia* transmission, it alone cannot explain the high number of infected arthropod species (Vavre et al., 1999; Werren et al., 2008). Horizontal transfers between hosts of different species provide a means for *Wolbachia* to spread to new hosts, and such cases have been reported in several systems (Ahmed et al., 2015; Baldo et al., 2008; Russell et al., 2003; Schuler et al., 2013, 2016; Zug & Hammerstein, 2018). Successful horizontal transmission of *Wolbachia* usually involves/requires (a) close physical contact between two species through shared parasitoids (Ahmed et al., 2015), cannibalism (Le Clec'h et al., 2013), plant‐mediated substrates (Li et al., 2017), or hybridization between closely related species (Cooper et al., 2019; Turelli et al., 2018); (b) the ability of *Wolbachia* to survive and proliferate within the new host (Sanaei et al., 2020); and (c) the ability to reach the germline, allowing it to be vertically transmitted and spread in the new host population (Toomey et al., 2013). Despite its importance for understanding *Wolbachia* evolution, examples of recent horizontal *Wolbachia* transfer in nature are scarce (Schuler et al., 2013).

Recent introductions of insects to new areas provide potentially fruitful systems to investigate horizontal *Wolbachia* transfer. Species introductions are usually viewed through the lenses of conservation biology (invaders disrupting the community structure of native species causing population decline or extinction) (Clavero et al., 2009; Gurevitch & Padilla, 2004), food web dynamics (invaders changing the trophic interactions among taxa in a manner altering the flow of energy through ecosystems) (Kimbro et al., 2009; Wolfe et al., 2008), and/or rapid evolutionary change (including ecological adaptation to new conditions, and even speciation) (Mooney & Cleland, 2001; Prentis et al., 2008). Studies of invasive species also usually focus on the changes caused by or to the introduced taxon (Herms & McCullough, 2014; Strayer et al., 1999). However, often overlooked, invasive species can also bring other associated organisms along with them, including parasites and microorganisms (Lee et al., 2020). One area that has been underexplored in this regard concerns the impact of invasive species on dynamics of the microbiome and, in particular, endosymbionts such as *Wolbachia*. Invasions set up conditions for the possible horizontal transfer of *Wolbachia*, especially for insects given their generally high infection rates. In principle, such transfers could occur in either direction from introduced to native host species or in the reverse direction from a native to an introduced host. In either case, evidence for horizontal transfer could be provided by comparative DNA sequence analysis of *Wolbachia* strains present in the introduced and natal home ranges of the invasive insects (e.g., Ahmed et al., 2016; Cooper et al., 2019).

Establishing a recent horizontal transfer can be quite informative for helping resolve the relative importance of different factors associated with a strain's transfer and subsequent spread (e.g., CI, resistance to phages/microbes, and/or increased fecundity or survivorship of the host). In essence, one could study the dynamics of the interaction and spread of a newly acquired *Wolbachia* after an initial horizontal transfer to monitor the chronology of different processes and factors contributing to its successful establishement in a new species. In addition, recent horizontal transfers provide a window into examining the dynamics of genome evolution occurring in *Wolbachia*. In this regard, recent comparative studies have revealed that the genome architecture of *Wolbachia* appears very dynamic (Klasson et al., 2008; Wu et al., 2004). Numerous instances of gene function gains and losses have been documented in different *Wolbachia* supergroups, as well as in CI inducing strains (LePage et al., 2017; Martinez et al., 2021; Shropshire & Bordenstein, 2019). Also, structural changes involving insertions, deletions, lateral transfers between coinfecting strains, and rearrangements appear to be common leading to mosaic‐like genomes (Klasson et al., 2009). Moreover, intragenic recombination has been shown in the standard sequencing method of multilocus sequence typing (MLST), which involves screening five *Wolbachia* genes for variation (*gatB*, *coxA*, *hcpA*, *fbpA*, *ftsZ*), resulting in phylogenies that do not necessarily reflect the evolutionary relationship between analysed strains (Baldo et al., 2006; Bleidorn & Gerth, 2018). As a consequence, these studies have called into question whether the MLST approach has sufficient power to resolve closely or even moderately diverged *Wolbachia* strains from one another.

The *Rhagoletis* cherry fruit fly system has emerged as an important model for studying *Wolbachia* population dynamics in nature (Baković et al., 2018; Boller et al., 1976; Riegler & Stauffer, 2002; Schuler et al., 2016). *Rhagoletis cerasi* (L.), is a common cherry‐infesting fruit fly native to Europe. Previous studies have reported that *R*. *cerasi* is infected with at least five distinct *Wolbachia* strains designated *w*Cer1 to *w*Cer5 found in various combinations and frequencies among populations (Arthofer et al., 2009; Riegler & Stauffer, 2002). In comparison, the CI inducing strain *w*Cer2 has been shown to cause up to 98% egg mortality in crosses between infected males and uninfected females (Boller & Bush, 1974). This strain is spreading northward through Europe in *R*. *cerasi* populations (Baković et al., 2018; Riegler & Stauffer, 2002; Schebeck et al., 2019; Schuler et al., 2016).

**FIGURE 1:**
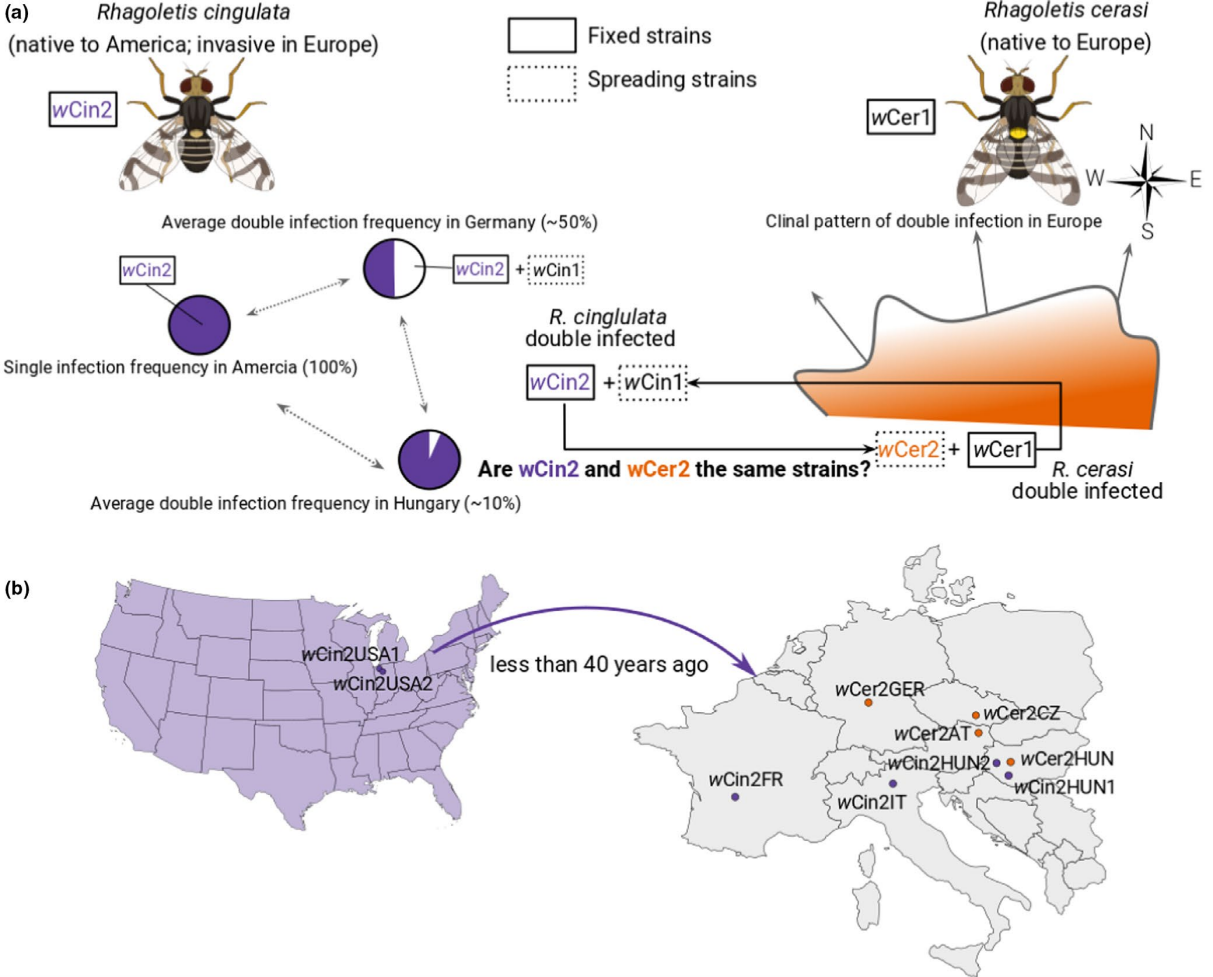
Cherry fruit fly study system. (a) Schematic representation of the infection status of *Rhagoletis cingulata* and *Rhagoletis cerasi* in Europe. *Rhagoletis cingulata*, native to North America, is universally infected with the *w*Cin2 *Wolbachia* strain (purple) present in every fly in the species. Pie charts depict the infection frequency of *R. cingulata*, ranging from high double infection (wCin2 + wCin1; white) in northern European populations to low double infection in south‐eastern populations. It remains unclear how connected are these populations (dotted arrows). *Rhagoletis cerasi*, native to Europe, is universally infected with the *Wolbachia* strain *w*Cer1, whereas most southern and central European populations are infected with an additional strain (*w*Cer2, orange). According to MLST and *wsp*, *w*Cer2 is identical to *w*Cin2 and hypothesized to have originated via a horizontal transfer from *R. cingulata*. (b) In Europe, *R. cingulata* is invasive and was introduced from America approximately 40 years ago. The dot represents the samples used in this study, where all *R. cingulata* (purple) are singly infected with *w*Cin2 and all *R. cerasi* (orange) are infected with wCer2 and *w*Cer1

*Rhagoletis* *cingulata* (Loew) is a cherry fruit fly native to North America, where it attacks the fruits of several cherry species (Boller et al., 1976; Bush, 1966; Doellman et al., 2020). Sometime in the late 20th century (ca. 40 years ago), *R*. *cingulata* was introduced to Europe, where the fly has rapidly spread and has since been reported in several European countries (Bjelis et al., 2016; Egartner et al., 2010; Merz & Niehuis, 2001; Szeőke, 2006). *R*. *cingulata* co‐occurs with *R*. *cerasi* over a significant portion of its invasive range in Europe, and the two species share cherry hosts in common. Thus, *R*. *cingulata* and *R*. *cerasi* fulfill some of the important host criteria conducive to the horizontal transfer of *Wolbachia*, as discussed above. Previous studies based on MLST sequencing identified two *Wolbachia* strains in *R*. *cingulata* (Drosopoulou et al., 2011; Schuler et al., 2009). The strain *w*Cin1 in *R*. *cingulata* was found to be essentially identical to *w*Cer1 in *R*. *cerasi* based on MLST markers and *wsp* and is present at different frequencies only in invasive *R*. *cingulata* populations in Europe and not found in its native range in North America (Figure 1a). This suggests a recent horizontal transfer event occurred in which *w*Cer1 from *R*. *cerasi* was acquired by *R*. *cingulata* in Europe within the last 40 years (Schuler et al., 2013). Since distantly related *Rhagoletis* species are unlikely to hybridize (Hood et al., 2012; Smith & Bush, 1997) and since no introgressed mitochondrial DNA has been observed it is unlikely that *Wolbachia* was horizontally transferred via hybridization (Schuler et al., 2013).

In contrast, all *R*. *cingulata* individuals in both North America and Europe have been found to harbour an additional strain *w*Cin2 which appears based on MLST sequencing to be identical to the strain *w*Cer2 in *R*. *cerasi* that is currently spreading through Europe (Schuler et al., 2013). This suggests that a bidirectional transfer of *Wolbachia* strains has occurred recently between invasive and native cherry fly host species in Europe. However, there are some inconsistencies concerning the bidirectional horizontal transfer hypothesis, particularly with regard to the transfer of *w*Cin2 from *R. cingulata* to *R. cerasi*. Specifically, there is evidence suggesting that *w*Cer2 may have invaded and been spreading in *R. cerasi* populations prior to the first report of *R. cingulata* in Europe (Boller et al., 1976; Riegler & Stauffer, 2002). Moreover, sequence analysis inferring that *w*Cer2 is essentially identical to *w*Cin2 is solely based on the classic MLST system (Baldo et al., 2006). However, as discussed above, recent comparative genome studies have shown that the MLST system may have limited power discriminating between closely related *Wolbachia* strains and should be augmented by whole‐genome sequencing approaches when possible to confirm stain identity (Bleidorn & Gerth, 2018; Scholz et al., 2020).

Here, we employ population‐level whole‐genome sequencing of *w*Cin2 and *w*Cer2 to revisit questions of horizontal strain transfer and *Wolbachia* strain diversity in the *Rhagoletis* cherry fruit fly system. We report striking differences between *w*Cin2 and *w*Cer2 that include gene gain and loss, genome structural differences, and variation in cytoplasmic incompatibility factor (*cif*) genes. Together, these differences discount the horizontal transfer hypothesis for these two strains and extend the literature about large‐scale changes in genome structure and diversification in *Wolbachia*.

## 2 MATERIALS AND METHODS

### 2.1 Fly collection and material maintenance

Fly collections were performed from 2016 to 2020 across Europe and in the United States (USA) (Figure 1b, Table S1). All flies were collected as larvae from infested cherry fruit and reared in the laboratory following standard *Rhagoletis* husbandry techniques (Tadeo et al., 2015). Larvae were either reared to the pupal stage for sequencing or were overwintered as pupae and collected after they eclosed as adults for sequencing. Samples that were not immediately used for sequencing were stored at either –80°C or in 100% EtOH at –20°C for later analysis.

### 2.2 Multiple displacement amplification and PacBio sequencing

Four samples were sequenced using the multiple displacement amplification (MDA) method (Ellegaard et al., 2013). (Table S1). For this approach, fly pupae were first homogenized using a plastic disposable pestle in phosphate buffer solution (PBS). *Wolbachia* were purified from pupae using multiple centrifugation steps and a syringe filtering step. We then amplified the bacterial pellet using a Repli‐G Midi kit (Qiagen). Amplified DNA was evaluated for quality and quantity using a NanoDrop (ThermoFisher). PacBio library preparation and sequencing was performed on a Sequel II platform at the Vienna Biocenter Core Facilities (VBCF). Additionally, 150 base pair (bp) paired‐end Illumina libraries were sequenced for the same MDA and PacBio sequenced samples. Illumina library preparation and sequencing were performed at the VBCF. To ensure that all *R. cerasi* samples are infected with *w*Cer2, we performed a diagnostic PCR with *w*Cer2‐specific primers (Riegler & Stauffer, 2002) prior to sequencing.

### 2.3 DNA extractions and Nanopore sequencing

Six samples were sequenced on the Oxford Nanopore (ONT) MinION platform (Table S1). For Nanopore sequencing, high molecular weight fly and *Wolbachia* DNA were extracted from six individual specimens using a high salt, SDS, and proteinase K lysis buffer, followed by two chloroform washes, and ethanol precipitation (see: protocols.io). Extracted DNA was evaluated for purity and quantity on a Nanodrop (ThermoFisher) and Qubit (ThermoFisher) fluorometer and purified as necessary using Ampure beads (Beckman Coulter). Sequencing libraries were prepared for each sample using the Oxford Nanopore ligation kit (SQK‐LSK109) and sequenced on R9.4.1 MinION flow cells. Bases were called using Guppy v4.2.2 with the HAC model. To improve the accuracy of our assemblies, Illumina 150 bp paired‐end libraries were sequenced for the same samples at the University of Notre Dame Genomics and Bioinformatics Core Facility (Indiana, USA).

### 2.4 Genome assembly and annotation

We assembled three *Wolbachia* *w*Cin2 genomes: two genomes for *Wolbachia* infecting *R. cingulata* from the native range of the fly in the USA (*w*Cin2USA1 and *w*Cin2USA2) and one genome for *Wolbachia* infecting *R. cingulata* from the invasive range in Europe (*w*Cin2HUN2). Reads were first mapped to the *w*Mel reference genome (GCF_000008025.1) (Wu et al., 2004) using minimap2 v2.17 (Li, 2018) to remove possible contaminants. Assemblies were generated using two long‐read only assemblers shown to produce highly accurate and contiguous assemblies (Wick & Holt, 2020): flye v2.8 (Kolmogorov et al., 2019) and canu v2.1.1 (Koren et al., 2017), with parameters optimized for assembling small bacterial genomes. We reviewed each assembly, and for each sample selected the most contiguous assemblies for polishing. All assemblies were first polished with long reads using racon v1.4.13 (Vaser et al., 2017). Nanopore assemblies were further polished using medaka v1.13. Finally, short reads were mapped to the assemblies using BWA‐MEM (Li, 2013). pilon v1.23 (Walker et al., 2014) was used to fix small indels. Assemblies were evaluated with busco v4.1.4 (Simão et al., 2015) to assess their completeness, using the Rickettsiales data set as a standard. All sequences and the new *w*Cin2 reference assembly (*w*Cin2USA1) were uploaded to GenBank (PRJNA713538 and assembly accession number CP072012).

We annotated the new *w*Cin2 genome (*w*Cin2USA1), and the previously published *w*Cer2 reference genome (GCA_011090435.1) using PGAP:2020–07–09.build4716 (Haft et al., 2018) and prokka v1.14.6 (Seemann, 2014). In addition to outputting a format directly submittable to NCBI, PGAP also produces annotations with fewer genes described as hypothetical. The PGAP annotation was kept for submission to the NCBI database (Accession number CP072012), while the Prokka annotation was used for downstream analysis as most tools conform to the gff format with unique gene IDs.

### 2.5 SNP calling and strain phasing

Because we were unable to produce high‐quality genome assemblies for all of our 10 sample genomes (Table S1), we used a two‐level phasing approach to characterize SNPs within *w*Cin2 and *w*Cer2. Long reads sequenced with PacBio and with ONT from both strains were mapped against the *w*Cin2USA1 or *w*Cer2 reference genomes (Morrow et al., 2020) with minimap2 v2.17. Duplicate reads were masked with samtools v1.10 (Li et al., 2009) and downsampled to a coverage limit of 50× to avoid excessive computational time. SNPs were called with freebayes v1.3.2 (Garrison & Marth, 2012). To ensure accurate variant calls, a Phred mapping quality score threshold of 30 and a base quality score of 20 was used. Additionally, SNPs were only accepted if they were present in more than five reads. whatshap v1.0 (Martin et al., 2016) with a maximum coverage per sample of 15× was used for a first round of phasing to leverage long read linkage information. shapeit v4.1.3 (Delaneau et al., 2019) was used for a second round of phasing to leverage conditional phase information contained across different populations. The mapping, SNP calling, SNP filtering, and phasing allowed us to filter out potentially coinfecting strains in *R. cerasi* (Arthofer et al., 2009), which according to Morrow et al. (2020) are less abundant than *w*Cer2.

### 2.6 Comparison of MSLT versus whole‐genome phylogenies

We first generated a MLST haplotype network for *w*Cin2 and *w*Cer2 for comparison to whole‐genome approaches. *Wolbachia* MLST is usually based on five housekeeping genes (*ftsZ*, *hcpA*, *fbpA*, *gatB*, *coxA*) and the hypervariable *wsp* (Baldo et al., 2006; Braig et al., 1998). The sequences *ftsZ* (JX073687.1, KJ546857.1), *hcpA* (JX073689.1, KJ546853.1), *fbpA* (JX073685.1, KJ546849.1), *gatB* (JX073691.1, KJ546845.1), *coxA* (JX073683.1, KJ546841.1), and *wsp* (JX073681.1, AF418557.1) were downloaded from NCBI for both *w*Cin2 and *w*Cer2 respectively (Arthofer et al., 2011; Riegler & Stauffer, 2002; Schuler et al., 2013). Using the downloaded sequence fragments, corresponding gene sequences in both the *w*Cin2 (*w*Cin2USA1) and *w*Cer2 reference genomes were identified and extracted with nucleotide BLAST searches and BEDTools (Quinlan & Hall, 2010). Sequences were then generated for each sample by using vcf2fasta (Sanchez‐Ramirez, 2020) to integrate sample‐specific phased SNPs into the gene sequences from the corresponding reference genome. Each sample had two sequences for each gene, one for each phase. These genes were aligned with mafft v7 (Katoh & Standley, 2013). Because the MSLT sequences are subsets of the full annotated genes, Gblocks online (Talavera & Castresana, 2007) was used to extract the aligned section of the genes. These aligned blocks were concatenated and visualized in seaview v4.0 (Gouy et al., 2010). Finally, raxml v8.2.12 (Stamatakis, 2014) with a GTRGAMMA model and 1000 bootstraps was used to make an unrooted phylogenetic tree for the MSLT loci.

With the same 10 *w*Cin2 and *w*Cer2 genomes used above for the MLST network, we then employed phased SNPs to construct a whole‐genome scale network. A pangenome for *w*Cin2 and *w*Cer2 was generated using panaroo v1.2.5 (Tonkin‐Hill et al., 2020). For each sample, sample‐specific phased SNPs were integrated into the pangenome with vcf2fasta creating two pangenomes for each sample, one for each phase. The pangenomes were then aligned with MAFFT and an unrooted phylogenetic tree was constructed using RaxML with a GTRGAMMA model and 1,000 bootstraps.

To discern higher‐order relationships to other *Wolbachia* strains, a rooted phylogenetic tree was made using A and B *Wolbachia* supergroup reference genomes downloaded from the NCBI RefSeq database (Table S2). The downloaded reference genomes, *w*Cin2 genomes (*w*Cin2USA1, *w*Cin2USA2, and *w*Cin2HUN2), and *w*Cer2 reference genome were first reannotated with Prokka. Panaroo was then used to identify a set of 202 core genes present in all samples. A rooted phylogenetic tree was generated using raxml v8.2.12 with a GTRGAMMA model and 1,000 bootstraps. To improve resolution of our phylogeny, we constructed a second rooted phylogenetic tree with reference *Wolbachia* only found within the *w*Mel complex in supergroup A using a set of 943 shared core genes. Again, a rooted phylogenetic tree was generated using RAxML with a GTRGAMMA model and 1000 bootstraps.

To date the divergence of *w*Cin2 and *w*Cer2, we constructed a chronogram for *w*Cin2USA1, *w*Cin2HUN2, and the reference wCer2 genomes. First, a core gene alignment was made with Panaroo and was partitioned by codon. The chronogram was generated using beast2 (Bouckaert et al., 2014) with linked trees, a yule tree model with default priors, strict molecular clocks for each partition, and with best fit substitution models selected with bmodeltest for each codon partition (Bouckaert & Drummond, 2017). We used a general *Wolbachia* estimate of 6.87 × 10^−9^ substitutions per third‐position site per year to calibrate the third codon position molecular clock (Richardson et al., 2012; Turelli et al., 2018). The analysis was run for 50 million generations, sampling every 5000 steps. When complete, convergence was checked, making sure ESS values were >200.

### 2.7 Genome comparisons

Phages were annotated with the PHASTER online tool (Arndt et al., 2016) on both our new reference *w*Cin2 (wCin2USA1) and the published *w*Cer2 reference (Morrow et al., 2020). mummer v3.0 (Marçais et al., 2018) was used to characterize repetitive sequences in the *w*Cin2 genome. lastal v1060 (Kiełbasa et al., 2011) was used to align *w*Cin2 and *w*Cer2 genomes against one another. For genome comparison, we extracted aligned blocks larger than 10,000 bp from the tab alignment file, and used circos v0.69–8 (Krzywinski et al., 2009) to visualize the collected information about GC‐content, repeats, SNP density in *w*Cin2, phages, and genes locations (with a particular focus on the MSLT and *cif* genes).

Finally, to compare gene presence and absence among cherry fly and the *w*Mel reference genomes, Panaroo was used to generate a 1402 gene pan‐genome. The VennDiagram package was used to visualize shared genes (Chen & Boutros, 2011) in r (R Core Team, 2019). For the genes unique to *w*Cin2, we extracted their corresponding protein sequences from the Prokka annotation files. Because the Prokka annotation is incomplete, we also manually curated these sequences using BLASTp searches to the NCBI microbial protein database and selected the best matching hit for each sequence.

## 3 RESULTS

### 3.1 Whole‐genome sequencing of wCin2

*Wolbachia* strains from *R. cingulata* and *R. cerasi* across Europe and the USA were sequenced using both PacBio and Oxford Nanopore sequencing technology (Table S3). Three high quality genomes were assembled: two *w*Cin2 genomes from the USA derived from a population of *R. cingulata* in Mishawaka, Indiana (designated *w*Cin2USA1 and *w*Cin2USA2) and one *w*Cin2 genome derived from a European population of *R. cingulata* in Hungary (designated *w*Cin2HUN2; Table S4). The best quality assembly, *w*Cin2USA1, was a continuous and circular 1.54 Mb genome, with a BUSCO score of 99.5% calculated using the Rickettsiales data set. The *w*Cin2USA1 assembly was subsequently used as the reference *w*Cin2 *Wolbachia* genome for all of the analyses that follow.

### 3.2 MLST based comparison of wCin2 and wCer2

**FIGURE 2:**
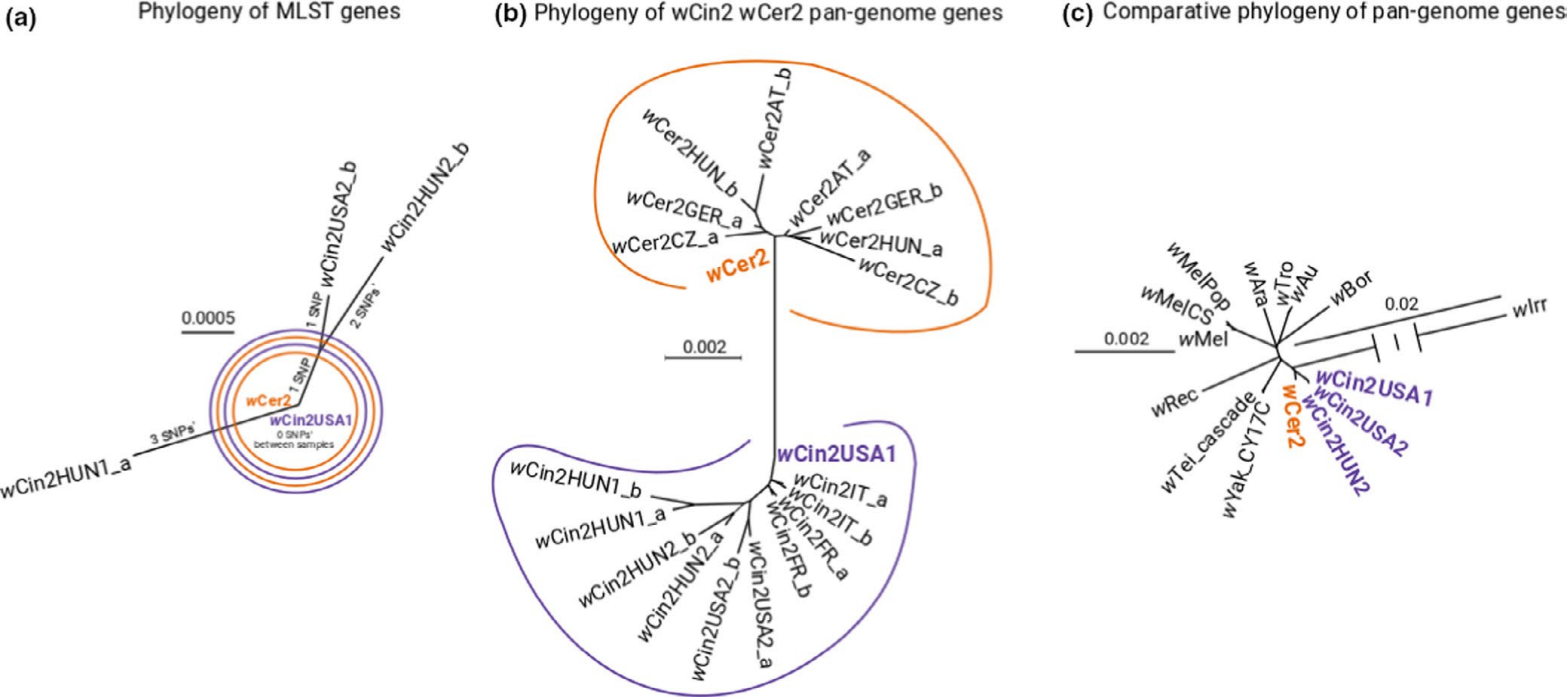
(a) An unrooted phylogenetic tree based on the six MLST markers for the four *w*Cin2 and six *w*Cer2 genomes sequenced in the current study, as well as the previous sequenced reference genome for *w*Cer2 (Morrow et al., 2020). Note there was no variation among the sequenced *w*Cer2 genomes for the six MLST loci and, thus they are all depicted in the network as *w*Cer2 in orange along with the reference genome. The reference genome for *w*Cin2 designated *w*Cin2USA1 is shown in purple. (b) An unrooted phylogenetic tree for the same set of genomes in panel a but based on 39,256 variable SNP sites across 1193 genes. The phasing approach leads to the split of each sequenced *Wolbachia* population into two major variants (subscript a and subscript b). (c) A rooted phylogenetic tree based on 943 shared core genes for cherry fruit fly reference genomes *w*Cin2 (purple) and *w*Cer2 (orange), along with reference genomes from other *w*Mel‐like *Wolbachia* from the *Wolbachia A* supergroup. For a rooted phylogenetic tree with b supergroup reference strains see Figure S1

The reference genomes *w*Cin1USA1 and *w*Cer2 were identical to one another based on sequence comparisons of the six MLST loci (Figure 2a). A total of seven SNPs were identified, however, from the MLST sequences and present in three of the six sampled *w*Cin2 genomes (*w*Cin2USA2, *w*Cin2HUN1, and *w*Cin2HUN2; Figure 2a). The North American *w*Cin2USA2 and Hungarian *w*Cin2HUN2 genomes shared one SNP distinguishing them from the other eight genomes sequenced in our population survey (Figure 2a). Five of the seven SNPs resided in the *cox*A gene, which is consistent with this locus generally displaying a high level of diversity in the *Wolbachia* genome (Baldo et al., 2006; Bleindorn & Gerth, 2018). No SNPs were detected among the six *w*Cer2 genomes sequenced with respect to the MLST loci. The results for the five MLST genes and the *wsp* gene analysed in the current study for 10 sampled genomes therefore concurred with the previous findings of Schuler et al. (2013) that *w*Cin2 and *w*Cer2 are essentially identical in sequence, consistent with the hypothesis of a recent horizontal transfer and spread of *w*Cer2 in European *R. cerasi* populations from a *w*Cin2 *R. cingulata* source. We did not find any evidence that characteristic SNPs for other *w*Cer strains such as *w*Cer1, *w*Cer3, *w*Cer4, *w*Cer5 (Arthofer et al., 2009) passed the filtering and phasing steps, suggesting that these strains were removed from the analysis due to their lower coverage as observed in Morrow et al. (2020).

### 3.3 Comparison of whole‐genome versus MLST phylogenies

The unrooted phylogeny for the whole‐genome data set constructed from 1193 homologous, single copy genes differed from the phylogeny based on the MSLT markers and showed clear divergence between the strains *w*Cin2 and *w*Cer2 (compare Figure 2a with 2b), refuting the horizontal transfer hypothesis. Within *R. cingulata*, there was little variation between phased *w*Cin2 genomes sequenced from the same individual, as both phases clustered together for each individual (Figure 2b). Lineages of *w*Cin2 may thus correspond or be constrained to matrilineages of flies in *R. cingulata*. In contrast, within *R. cerasi*, the phased *w*Cer2 genomes present in the same individual differed and were more similar to a genome found in a different fly elsewhere in Europe than to the alternate phased genome in the same individual (Figure 2b).

The rooted phylogeny constructed with 943 shared core genes using reference genomes from other closely related *w*Mel‐like *Wolbachia* strains further supported that *w*Cin2 and *w*Cer2 are distinct *Wolbachia* A supergroup strains (Figure 2c, see Figure S1 for a rooted phylogeny including B type strains). As before, variation between *w*Cin2 genomes was substantially less than the level of divergence between *w*Cin2 and *w*Cer2, in this case involving a comparison between the three reference *w*Cin2 genomes initially constructed here (*w*Cin2USA1, *w*Cin2USA2, and *w*Cin2HUN2) vs. the previously published *w*Cer2 reference genome of Morrow et al. (2020). Nevertheless, *w*Cin2 and *w*Cer2 were more closely related to each other than to any other *w*Mel‐like *Wolbachia* strain.

### 3.4 Comparative genomics of wCin2 and wCer2

**FIGURE 3:**
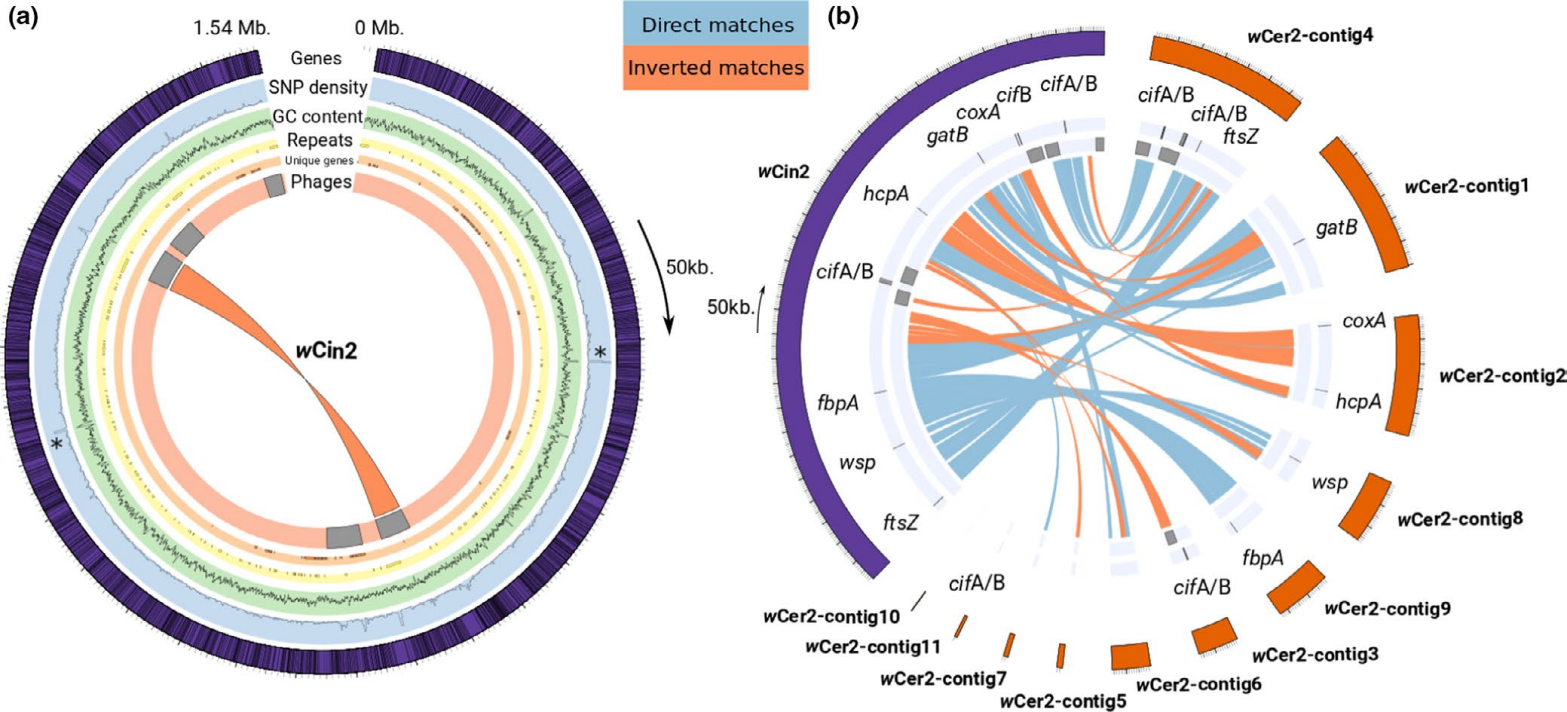
(a) CIRCOS plot for the *w*Cin2 reference genome *w*Cin2USA1, with a total length of ~1.54 Mb. The outer track of the CIRCOS plot in purple represents gene density. Every gene is represented by a line on the track. The next inner track in light blue represents SNP density and the third track in green is GC‐content. Asterisks denote two regions where high GC‐content is associated with high SNP‐density. The fourth track in yellow shows the distribution of repetitive elements and the fifth track in pink shows the unique genes present in *w*Cin2 but not found in *w*Cer2 or in *w*Mel (see Figures S2 and S3; Table S6 for additional details). In the sixth and innermost track, phage positions are plotted as grey blocks. We found five complete phages of which two are inverted duplicates of each other (see red band). (b) Comparison between the *w*Cin2USA1 (in purple; 1.54 Mb) and the *w*Cer2 (in orange; 1.33 Mb) reference genomes. The first outermost track shows the location of the six MLST genes, and the *cif*A, and *cif*B cytoplasmic incompatibility factors. The second track in light blue highlights the phage regions. The blue bands between both genomes show the direct matching regions larger than 10 kb oriented in the same direction. In orange, the bands depict inverted regions between the two genomes

The *w*Cin2 reference genome (*w*Cin2USA) was comprised of 1,520 identified genes together constituting 1.3 Mb or ~84% of the total genome length (Figure 3a). The average GC‐content of the *w*Cin2 reference genome was 35.1% (range 24.5% to 52.9%). Mean SNP density among the four sequenced *w*Cin2 genomes was 4.6 per 1000 bp, with a maximum of 77 SNPs per 1000 bp in one region. The region of highest SNP‐density, along with one other high SNP‐density region, were both associated with higher GC‐content (see asterisks in Figure 3a). There was a total of 18,745 variable sites in the gene space across all 10 *w*Cin2 and *w*Cer2 genomes, corresponding to a mean of about 1.8 polymorphisms per hundred bp.

Although the *w*Cer2 genome was fragmented in 11 contigs, we could still identify multiple major inversions between *w*Cer2 and *w*Cin2, particularly in *w*Cer2‐contig2 where both the *hcpA* and the *coxA* genes are positioned (Figure 3b; Table S5). Repetitive elements were widespread and uniformly distributed across the *w*Cin2 genome. There were five intact phages identified in the *w*Cin2USA1 reference genome, as annotated by PHASTER. Two of these phages were inverted duplicates of each other, suggesting that these phages were not acquired from another *Wolbachia* but instead were duplicated within the *w*Cin2 strain (see red bands in Figure 3a). In contrast, *w*Cer2 has a single complete phage along with two incomplete phages (Morrow et al., 2020), providing further evidence for major differences in genome content, organization, and patterning between *w*Cer2 and *w*Cin2. In addition to these structural differences, the number of *cif* genes, causal factors associated with CI of phage origin, differed between *w*Cin2 and *w*Cer2 (Figure 3b). In addition to finding the three *cif*A/B tandems and the isolated type V *cif*B in *w*Cer2 described by Morrow et al. (2020), we found an additional undescribed type V *cif*A associated with the isolated type V *cif*B. This brought the number of *cif*A/B tandems in *w*Cer2 to four. We did not find any isolated *cif*B genes in *w*Cer2. In contrast, we found two *cif*A/B tandems in *w*Cin2 and one isolated *cif*B (Figure 3b). Because the copy number of *cif* genes seems to correlate with the strength of CI in other *Wolbachia* genomes (LePage et al., 2017), *w*Cer2 might cause higher strength of CI than *w*Cin2.

Taken together, the above results suggest that there has been a major genomic expansion within *w*Cin2, especially in comparison to *w*Cer2 and other closely‐related *Wolbachia* strains. Strain *w*Cin2 was found to have a larger genome (1.54 Mb) and more annotated genes (1520) compared to both *w*Cer2 (1.33 Mb, 1260 annotated genes) and the rest of the *w*Mel *Wolbachia* core group (mean for genomes used in our study: 1.25 Mb, 1094 annotated genes; see Table S2 for size comparisons). Gene presence and absence for the 1402 pan‐genome of *w*Cin2 (*w*Cin2USA1), *w*Cin2HUN2, *w*Cer2, and *w*Mel confirmed that *w*Cin2 has undergone dramatic genome expansion, although some caution is warranted due to the fragmented *w*Cer2 assembly and the possibility of misassembled repetitive regions (Figure S2). The genomes wCin2USA1 and *w*Cin2HUN2 were found to possess 175 unique genes not shared with *w*Cer2 and *w*Mel. In comparison, the *w*Mel genome contained 33 unique genes and *w*Cer2 40 unique genes. Unlike repeatswhich were uniformly distributed across the *w*Cin2 genome, these unique genes tended to cluster together within the *w*Cin2 genome (Figure 3b). Manual curation of these unique 175 *w*Cin2 genes using a BLASTp search of the NCBI microbial database found 88 uncharacterized or hypothetical proteins and 87 proteins with associated annotations. Of the 87 proteins with known annotations, phage associated proteins made up 39% of the unique genes, and transposase and recombinase proteins make up an additional 15% of the novel genes (Figure S3; Table S6). Together, a major faction of the identified unique genes found in *w*Cin2 are associated with WO‐phage and their clustering suggest that these genes might be portions of phages missed by PHASTER (Bordenstein & Bordenstein, 2016; Miao et al., 2020).

## 4 DISCUSSION

Recent species invasions provide real‐time natural experiments for investigating the ecological and evolutionary dynamics accompanying exposure to new biotic and abiotic interactions, and selective pressures. One facet of species introductions that has not been fully leveraged in this regard concerns the interactions between endosymbionts carried by invading host species and native endosymbionts present in resident hosts. Recent horizontal transfer events of a *Wolbachia* strain from one host to another represent the equivalent of a new species introduction and, thus, provide opportunities to study the ecological and evolutionary dynamics of endosymbiont interactions as they first occur and unfold with each other and with their hosts. Although horizontal transfer events of *Wolbachia* in nature are not uncommon (Cooper et al., 2019; Hill et al., 2020; Li et al., 2017), well‐documented cases of recent transfer are still rare. However, the possibility exists that when the host of an endosymbiont is introduced into a new area, this may increase the chances for *Wolbachia* to be transferred to new hosts that share the same ecological niches. This may be particularly relevant for *Wolbachia* endosymbionts that influence the fitness of their hosts and infect common insects.

Previous studies based on the sequencing of MLST markers implied that bidirectional horizontal transfer of *Wolbachia* have occurred between the cherry‐infesting fruit flies *R. cingulata* and *R. cerasi*, following the former species introduction from North America to Europe ~40 years ago. However, recent comparative genomics work has raised questions about the sensitivity of the standard MLST sequence approach for distinguishing among closely related or even moderately diverged strains of *Wolbachia* (Bleidorn & Gerth, 2018; Scholz et al., 2020). We therefore undertook a whole‐genome sequencing study to confirm the earlier MLST and *wsp* results for the cherry flies, focusing on one of the two hypothesized directions of horizontal transfer; from the universal *Wolbachia* strain *w*Cin2 infecting the introduced *R. cingulata* to the native *R. cerasi*, where the strain is designated *w*Cer2.

Our findings were both surprising and striking. Comparative genomics of *w*Cin2 and *w*Cer2 revealed that the two strains are quite different, discounting the recent horizontal transfer hypothesis, at least in the direction of *R. cingulata* to *R. cerasi* in Europe. Not only were the DNA sequences of a set of 1,193 homologous, single copy genes fairly diverged between *w*Cin2 and *w*Cer2 (1.8%) but we also identified major structural rearrangements, copy number variation in CI inducing genes, and the presence of several genes unique to the *w*Cin2 strain. All of these differences were detected by whole genome sequencing despite MLST sequences being essentially identical between the two strains. Our results therefore confirm the utility of whole‐genome sequencing and raise caution concerning the ability of standard MLST barcoding that focuses on portions of limited numbers of genes to assign *Wolbachia* strain identity. Indeed, our findings can be taken as a general warning to be careful in interpreting any barcoding approach that focuses on a portion of the genome (or on a genome) that might not capture fully all the evolutionary history of a taxon or group of taxa.

Our rejection of a recent horizontal origin of *w*Cer2 raises several questions concerning the source of this strain and the dynamics of its spread in *R. cerasi* across Europe. Under the horizontal transfer hypothesis, the history of *w*Cer2 could be accounted for by *R. cerasi* recently acquiring the strain from *R. cingulata*, with its CI‐associated effects contributing to the strains successful transfer and rapid spread through *R. cerasi* populations in Europe. But in light of our findings, this scenario must be reevaluated. It is still possible that *R. cerasi* recently acquired *w*Cer2 and the strain is rapidly spreading through Europe. However, the source of the strain was not *w*Cin2 from introduced *R. cingulata*. Sequences of wCin2 from *R. cingulata* specimens collected in Europe and the United States were essentially identical to one another, with the exception of some intrastrain variation present in *w*Cin2. Importantly, the lack of sequence differences in *w*Cin2 between flies from the two continents supports the natural history of a recent introduction of *R. cingulata* to Europe ~40 years ago. However, assuming a substitution rate in *Wolbachia* of 6.87 × 10^−9^ substitutions per third‐position site per year (Richardson et al., 2012; Turelli et al., 2018) a rough gauge of the divergence time of *w*Cin2 and *w*Cer2 would be ~137,206 years (110,943–179,758, 95% CI). Thus, if *R. cingulata* was the source of *w*Cer2 in *R. cerasi*, the transfer would have to have happened much more distantly in the past, possibly involving a previous and now extinct colonization by the fly of Europe. If true, this would imply that the spread of *w*Cer2 in *R. cerasi*, while occurring, is proceeding at a much slower pace than previously thought. Regardless, rejection of *w*Cin2 being recently horizontally transferred from *R. cingulata* now requires detective work involving whole‐genome sequencing of *Wolbachia* in other insects in Europe to help resolve the source of wCer2 in *R. cerasi*. In addition, detailed analysis of intrastrain variation in wCer2 is also needed to determine the age of association of the strain with the fly and better gauge its rate of spread. Our phylogenomic analysis of *Wolbachia* implied that A group *Wolbachia* from the *D. yakuba* complex are most closely related to *w*Cin2 and *w*Cer2 (Cooper et al., 2019). However, *w*Cin2 and *w*Cer2 were still more closely related to each other than to any other strain yet sequenced, indicating that much further work in this area is still needed to determine the source of *w*Cer2 in *R. cerasi*, including a detailed survey of other *Rhagoletis* taxa.

In regard to the issue of intrastrain variation, although over a thousand *Wolbachia* genomes have now been sequenced, there are few examples of natural population‐level whole‐genome sequencing data sets (Hill et al., 2020). Rare variants of the predominant *Wolbachia* strain have been described in previous studies in *Rhagoletis pomonella* (Schuler et al., 2011), *R. cerasi* (Schneider et al., 2013) and *R. cingulata* (Schuler et al., 2013). Here, our population‐level sequencing allowed us to confirm these previous studies and detect additional SNP variation across the genomes of *w*Cin2 and *w*Cer2 present in natural populations. Although we do not have a clear picture of *Wolbachia* lineage diversity in the *R. cingulata* native range, lineages of *w*Cin2 in Europe may be associated with a loss of diversity, possibly due to bottlenecks in the invasive range. In contrast, within *R. cerasi*, the phased *w*Cer2 genomes present in the same individual differed and were more similar to a genome found in a different fly elsewhere in Europe than to the alternate phased genome in the same individual. This finding suggests that *R. cerasi* flies may be commonly co‐nfected by different minor variants of *w*Cer2 which could have been cosegregating for a long time, or that *R. cerasi* might have acquired multiple similar cosegregating strains at the same time. Thus, although populations of *Wolbachia* strains can be closely related, they may be less monoclonal than previously believed. This highlights that besides detecting major differences between closely related *Wolbachia* strains in different species, comparative studies using whole‐genome sequencing also have the potential to permit fine‐scale analysis of the population structure of natural field populations. Thus, further whole‐genome sequencing studies expanding our survey of *w*Cer2 in European populations of *R. cerasi* could prove highly informative for clarifying the history and dynamics of the spread of the strain in the fly.

Our results for *w*Cer2 also raise questions about whether *w*Cin1 was acquired in European populations of *R. cingulata* by horizontal transfer of *w*Cer1 from *R. cerasi*. Given our finding that whole‐genome sequencing of *w*Cin2 and *w*Cer2 revealed significant differences between these two strains, while MLST analysis suggested the strains were the same, it is now imperative to perform whole‐genome sequencing of *w*Cin1 and *w*Cer1, as well, to assess their relationship. There are reasons to think that horizontal acquisition of *w*Cin1 is the most likely scenario for the origin of the strain in *R. cingulata* as *w*Cin1 has yet to be found in any population of the fly in its native range in North America (Schuler et al., 2013). Given the evidence from *w*Cin2 supporting the recent introduction of *R. cingulata* into Europe, the implication is that the fly had to obtain the new strain recently, as well. Thus, even if the source of *w*Cin1 was not *w*Cer1 from *R. cerasi*, then *R. cingulata* probably obtained the endosymbiont from some other host species in Europe. Consequently, despite the source species being in question, the case for horizontal transfer remains viable. Nevertheless, whole‐genome sequencing of *w*Cin1 and *w*Cer1 is still required before more definitive conclusions can be drawn.

### 4.1 Comparative genomics of wCin2 and wCer2

In addition to the analysis of sequence polymorphism and divergence, whole‐genome sequencing allows comparison of the composition and structure of genomes. Recent comparisons of whole‐genome sequences of *Wolbachia* have highlighted that strains can differ greatly in a number of ways besides just in the sequences of homologous genes. For example, the genomes of *Wolbachia* are highly dynamic with respect to the numbers and positions of repetitive elements, mobile genetic elements, and the genes they contain (Klasson et al., 2008; Wu et al., 2004). Here, we found that the *w*Cin2 genome contains a comparatively high number of phages and repetitive elements. Moreover, the *w*Cin2 genome is 16% larger than *w*Cer2 (however, this might be influenced by the fragmentation of this draft genome) and 23% larger than the average genome size of other reference strains included in this study. We identified 175 genes unique to *w*Cin2 compared to 40 unique genes in *w*Cer2, most of which code for phage and transposon elements, implying a genome in flux. In *w*Cin2, two of the five annotated phages were inverted duplicates (a rearrangement) of each other suggesting that at least one of these two elements replicated within *w*Cin2. Repeated and frequent population bottlenecks during *Wolbachia* transmission coupled with genetic drift are population‐level factors that can render selection inefficient in *Wolbachia*, resulting in the accumulation of repetitive elements and imposing high mutation loads (Moran, 1996). The 175 unique genes in *w*Cin2 suggest that many genomic regions in this strain could have been acquired (possibly recently) from outside sources, such as other *Wolbachia* strains, host nuclear DNA, or even other species, and their association with WO‐phages reinforces the notion that the mobilome plays an important role in *Wolbachia* strain differentiation (Bordenstein & Bordenstein, 2016; Bordenstein et al., 2006; Brown & Lloyd, 2015; Rosenkranz et al., 2010).

The strains *w*Cin2 and *w*Cer2 also differed in another important way concerning the genes responsible for cytoplasmic incompatibility. The recent identification of two essential causal factors involved in CI, namely the *cifA* and *cifB* genes (Beckmann et al., 2017; LePage et al., 2017; Shropshire & Bordenstein, 2019), has prompted a number of studies to look into copy number variation and genetic variation at these loci to better understand the evolution of CI (Martinez et al., 2021), and the evolutionary relationships of *Wolbachia* strains (Cooper et al., 2019; Lindsey et al., 2018; Martinez et al., 2021; Turelli et al., 2018). The fact that CI loci occur in the eukaryotic association module of prophage WO has also brought significant attention to the role of phages as fast‐evolving features in *Wolbachia* genomes (Bordenstein & Bordenstein, 2016; Bordenstein et al., 2006; Tanaka et al., 2009) that can also promote horizontal gene transfer between strains (Wang et al., 2016). In the current study, different numbers of cytoplasmic incompatibility factors were found between *w*Cin2 and *w*Cer2. Most notably, two *cif*A/B complex were discovered in *w*Cin2 and four complexes in *w*Cer2 (Morrow et al., 2020) implying that these strains may cause different levels of CI or be bidirectionally incompatible with each other (Bonneau et al., 2018). However, future artificial transinfection studies are needed to discern the phenotypic effects of these two *Wolbachia* strains and equate strength of CI to the *cif* genes they possess.

## 5 CONCLUSION

The horizontal transfer of *Wolbachia* strains between hosts, although not an uncommon event, is difficult to catch in the act. One potentially recent instance of horizontal transfer inferred from sequencing MLST markers centred on the acquisition of a new strain of *Wolbachia* by European populations of the cherry fruit fly *R. cerasi* from the introduced *R. cingulata*. However, whole‐genome sequencing performed here showed that the genomes of the two strains are different in sequence, composition, and structure, yet essentially identical for MLST markers. Our results therefore discount the horizontal transfer hypothesis, support the difficulty of detecting recent transfer events, provide more evidence for extensive genome evolution among *Wolbachia* strains, and further underscore the need for whole‐genome sequencing to resolve different strains of *Wolbachia* from one another.

## AUTHOR CONTRIBUTIONS

Thomas M. Wolfe carried out DNA extractions and library preparation, bioinformatic analyses, fly collection, and manuscript writing. Daniel J. Bruzzese carried out DNA extractions and library preparation, bioinformatic analyses, fly collection, and manuscript writing. Erika Corretto did the library preparations for sequencing. Sonja Lečić contributed to bioinformatic analyses and the manuscript draft. Lisa Klasson contributed to bioinformatic analyses, and manuscript writing. Christian Stauffer contributed to the study design, fly collection, and scientific contributions. Jeffrey L. Feder contributed to the study design and scientific contributions. Hannes Schuler contributed to the study design, manuscript writing fly collection, overall supervision.

## Supporting information

Fig S1‐S3

Table S1‐S6

## Data Availability

The DNA extraction protocol for Nanopore sequencing is available at protocol.io (https://www.protocols.io/private/F7A32E6B312911EB952C0A58A9FEAC2A). The raw sequencing data for *w*Cer2 and *w*Cin2 were deposited on NCBI SRA (PRJNA712975 and PRJNA713538). The new *w*Cin2 assembly has been deposited on GenBank (CP072012). Data and scripts are available on Dryad (https://doi.org/10.5061/dryad.p8cz8w9pz).
