# Supplementary material for: Comparative genome sequencing reveals insights into the dynamics of Wolbachia in native and invasive cherry fruit flies
Source: Mol Ecol. 2021 May 7;30(23):6259–72. doi: 10.1111/mec.15923 (PMC9290052; doi:10.1111/mec.15923)

**Supplementary Figures and captions**

**
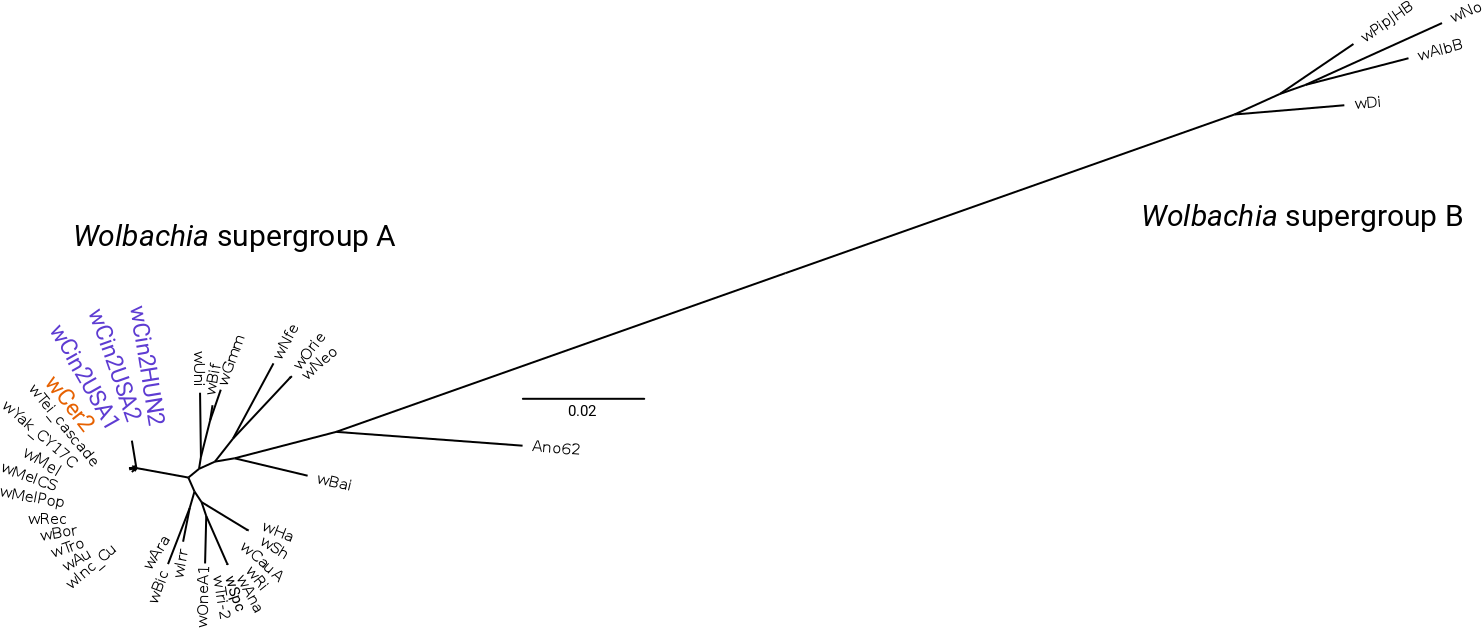
**

**Figure S1.** A rooted phylogenetic tree for *w*Cin2, *w*Cer2, and other *Wolbachia* reference genomes from supergroup A and supergroup B strains based on 202 shared core genes. Note that because of the limited number of shared core genes used to construct the network there was no resolution within the *w*Mel complex.


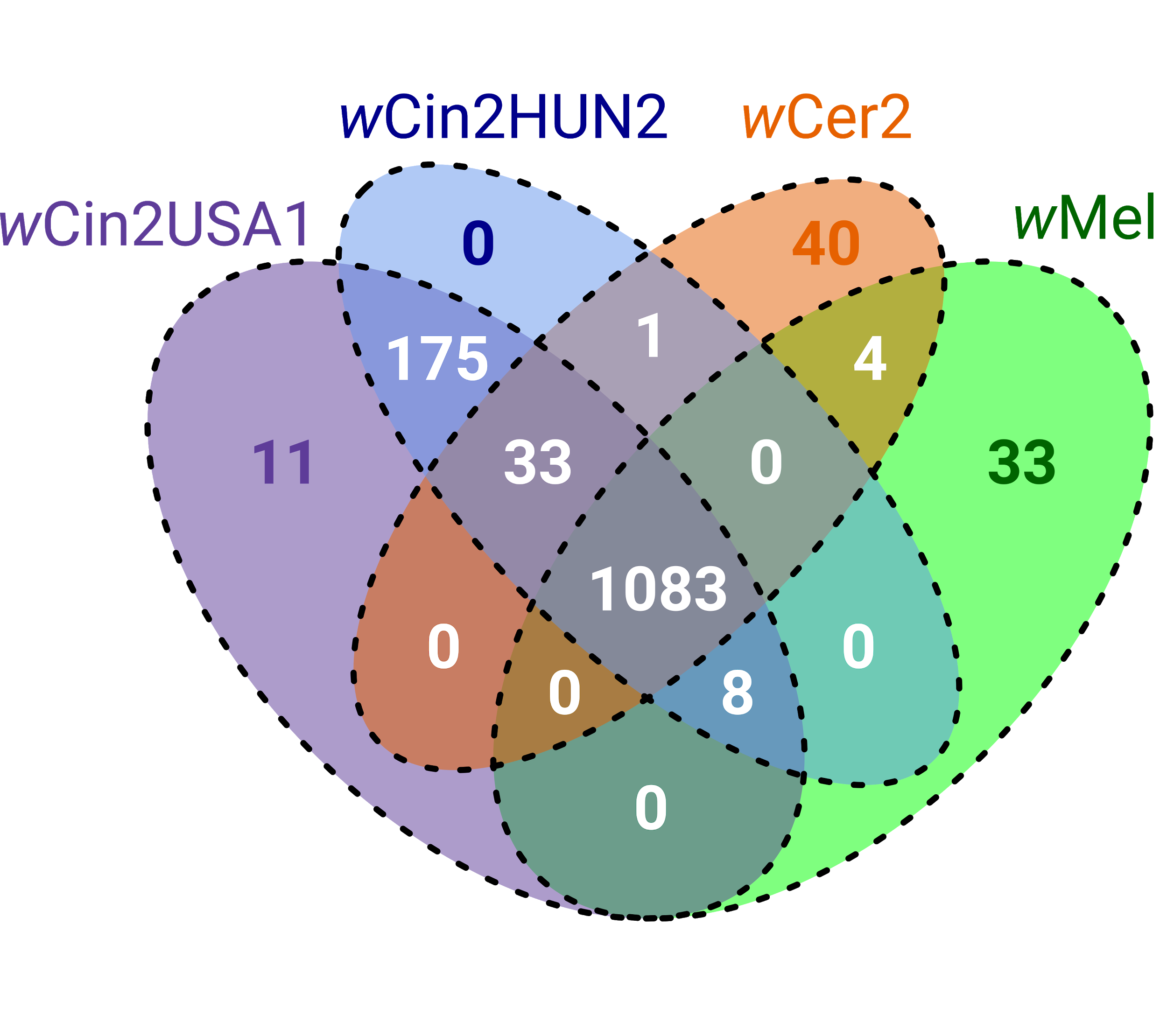


**Figure S2.** Venn overlap diagram for gene presence and absence for the 1,402 loci in the pan-genome of *w*Cin2USA1, *w*CinHUN2, *w*Cer2, and *w*Mel. Both *w*Cin2USA1 and wCin2HUN2 share 175 genes that are not found in wCer2.

**Figure S3.** Known functional categories from the NCBI microbial protein dataset for the 87 of 175 unique genes identified in the *w*Cin2 reference genome. The remaining half of 88 gene products were annotated as unknown or hypothetical and were not included. The category named other is composed of functional categories that have less than two occurrences.


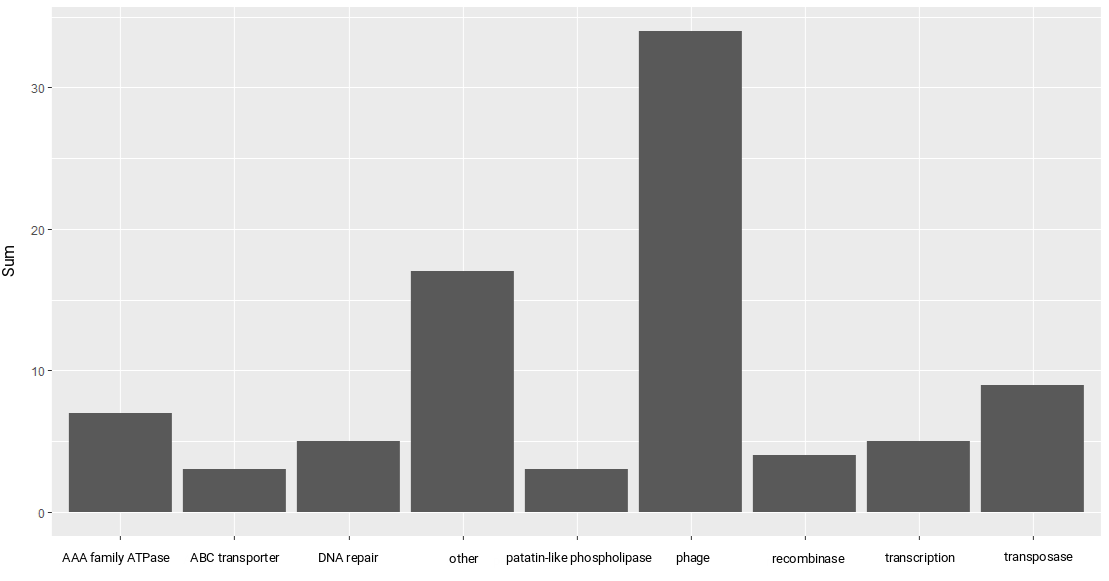

Supplement: Supplementary file 1 — Fig S1‐S3 [file MEC-30-6259-s002.docx]
