# Supplementary material for: Comparative genome sequencing reveals insights into the dynamics of Wolbachia in native and invasive cherry fruit flies
Source: Mol Ecol. 2021 May 7;30(23):6259–72. doi: 10.1111/mec.15923 (PMC9290052; doi:10.1111/mec.15923)
Supplement: Supplementary file 2 — Table S1‐S6 [file MEC-30-6259-s001.docx]

**Supplementary Tables**

| **Sample ID** | **species** | **country** | **Site** | **latitude** | **longitude** | **Year** | **MDA** | **Sequencing**  **platform** | **NCBI BioSample** |
| --- | --- | --- | --- | --- | --- | --- | --- | --- | --- |
| *w*Cin2USA1 | *R. cingulata* | USA | Mishawaka, Indiana | 41.697347 | -86.172172 | 2019 | No MDA | ONT  MinION | SAMN18341210 |
| *w*Cin2USA2 | *R. cingulata* | USA | Mishawaka, Indiana | 41.697347 | -86.172172 | 2019 | MDA | PacBio  Sequel II | SAMN18256848 |
| *w*Cin2HUN1 | *R. cingulata* | Hungary | Kaszó,  Hungary | 46.31594 | 17.223593 | 2018 | MDA | PacBio  Sequel II | SAMN18256849 |
| *w*Cin2HUN2 | *R. cingulata* | Hungary | Türje,  Hungary | 47.014844 | 17.08967 | 2018 | MDA | PacBio  Sequel II | SAMN18256850 |
| *w*Cin2FR | *R. cingulata* | France | Beynat, France | 45.1592086 | 1.7526254 | 2020 | No MDA | ONT  MinION | SAMN18256851 |
| *w*Cin2IT | *R. cingulata* | Italy | Calò,  Italy | 45.6735757 | 9.2564404 | 2020 | No MDA | ONT  MinION | SAMN18256852 |
| *w*Cer2CZ | *R. cerasi* | Czechia | Brno,  Czechia | 49.207818 | 16.599238 | 2019 | MDA | PacBio  Sequel II | SAMN18238200 |
| *w*Cer2HUN | *R. cerasi* | Hungary | Felsőörs, Hungary | 47.022416 | 17.922458 | 2018 | No MDA | ONT  MinION | SAMN18238201 |
| *w*Cer2AT | *R. cerasi* | Austria | Florisdorf,  Austria | 48.289778 | 16.436822 | 2019 | No MDA | ONT  MinION | SAMN18238202 |
| *w*Cer2GER | *R. cerasi* | Germany | Hailer,  Germany | 50.184922 | 9.164467 | 2016 | No MDA | ONT  MinION | SAMN18238203 |

**Table S1.** Ten sample genomes of strains *w*Cin2 and *w*Cer2 sequenced in the study. Given are the species, the country, location (site with latitude and longitude coordinates), collection year, and the sequencing platform used. The wCin2 reference genome was built from the singly infected sample from USA, wCin2USA1 (GENBANK ID).

| **Strain** | **grp** | **Genome_ID** | **Size(Mb)** | **Contigs** | **Reference** |
| --- | --- | --- | --- | --- | --- |
| wAlbB | B | GCF_004171285.1_ASM417128v1 | 1.48401 | 1 | (Sinha, Li, Sun, & Carlow, 2019) |
| wNo | B | GCF_000376585.1_ASM37658v1 | 1.30182 | 1 | (Ellegaard, Klasson, Näslund, Bourtzis, & Andersson, 2013) |
| wPipJHB | B | GCF_000156735.1_ASM15673v1 | 1.54366 | 16 | (Salzberg, Puiu, Sommer, Nene, & Lee, 2009) |
| wDi | B | GCF_013458815.1_ASM1345881v1 | 1.65629 | 1 | (Saha et al., 2012) |
| wAno62 | A | GCF_014333535.1_ASM1433353v1 | 1.20268 | 96 | (Lee, Lin, Tseng, Matsuura, & Yang, 2020) |
| wSpc | A | GCF_002300525.1_ASM230052v1 | 1.42034 | 106 | (Conner et al., 2017) |
| wRi | A | GCF_000022285.1_ASM2228v1 | 1.44587 | 1 | (Klasson et al., 2009) |
| wAna | A | GCF_008033215.1_ASM803321v1 | 1.40146 | 1 | (Gasser, Chung, Bromley, Nadendla, & Hotopp, 2019) |
| wTri-2 | A | GCF_014129515.1_ASM1412951v1 | 1.28954 | 9 | (Martinez, Klasson, Welch, & Jiggins, 2021) |
| wOneA1 | A | GCF_009012935.1_ASM901293v1 | 1.29341 | 47 | (Wang et al., 2019) |
| wCauA | A | GCF_006542295.1_ASM654229v1 | 1.44934 | 1 | Not published |
| wSh | A | GCF_014354315.1_ASM1435431v1 | 1.29489 | 83 | (Hague, Caldwell, & Cooper, 2020) |
| wBic | A | GCF_014129645.1_ASM1412964v1 | 1.18287 | 24 | (Martinez, Klasson, Welch, & Jiggins, 2021) |
| wOrie | A | GCF_014129565.1_ASM1412956v1 | 1.36398 | 19 | (Martinez, Klasson, Welch, & Jiggins, 2021) |
| wNeo | A | GCF_014129535.1_ASM1412953v1 | 1.35568 | 19 | (Martinez, Klasson, Welch, & Jiggins, 2021) |
| wNfe | A | GCF_001675785.1_ASM167578v1 | 1.33764 | 231 | (Gerth & Bleidorn, 2017) |
| wBai | A | GCF_014129605.1_ASM1412960v1 | 1.19096 | 26 | (Martinez, Klasson, Welch, & Jiggins, 2021) |
| wBif | A | GCF_014129685.1_ASM1412968v1 | 1.19024 | 17 | (Martinez, Klasson, Welch, & Jiggins, 2021) |
| wGmm | A | GCF_000689175.1_wGmm_version4 | 1.01951 | 201 | (Brelsfoard et al., 2014) |
| wUni | A | GCF_000174095.1_wUni_1.0 | 0.867873 | 256 | (Klasson et al., 2009) |
| wHa | A | GCF_000376605.1_ASM37660v1 | 1.2958 | 1 | (Ellegaard, Klasson, Näslund, Bourtzis, & Andersson, 2013) |
| wInc_Cu | A | GCF_001758565.1_ASM175856v1 | 1.26784 | 1 | (Wallau, da Rosa, De Ré, & Loreto, 2016) |
| wIrr | A | GCF_009732755.1_ASM973275v1 | 1.35235 | 1 | (Madhav, Parry, Morgan, James, & Asgari, 2020) |
| wMel | A | GCF_000008025.1_ASM802v1 | 1.26778 | 1 | (Wu et al., 2004) |
| wMelCS | A | GCF_014354335.1_ASM1435433v1 | 1.23379 | 89 | (Hague, Caldwell, & Cooper, 2020) |
| wMelPop | A | GCF_000475015.1_wMelPop | 1.23916 | 25 | (Woolfit et al., 2013) |
| wAu | A | GCF_000953315.1_Wau001 | 1.26846 | 1 | (Sutton, Harris, Parkhill, & Sinkins, 2014) |
| wTei_cascade | A | GCF_005862135.1_ASM586213v1 | 1.30133 | 122 | (Cooper, Vanderpool, Conner, Matute, & Turelli, 2019) |
| wYak_CY17C | A | GCF_005862115.1_ASM586211v1 | 1.28885 | 118 | (Cooper, Vanderpool, Conner, Matute, & Turelli, 2019) |
| wAra | A | GCF_014129655.1_ASM1412965v1 | 1.29323 | 10 | (Martinez, Klasson, Welch, & Jiggins, 2021) |
| wTro | A | GCF_014129525.1_ASM1412952v1 | 1.21575 | 13 | (Martinez, Klasson, Welch, & Jiggins, 2021) |
| wBor | A | GCF_014129615.1_ASM1412961v1 | 1.2142 | 16 | (Martinez, Klasson, Welch, & Jiggins, 2021) |
| wRec | A | GCF_000742435.1_ASM74243v1 | 1.126656 | 43 | (Metcalf, Jo, Bordenstein, Jaenike, & Bordenstein, 2014) |
| wCer2 | A | GCA_011090435.1_ASM1109043v1 | 1.325568 | 11 | (Morrow et al., 2020) |

**Table S2.** *Wolbachia* reference genomes used for constructing whole-genome phylogenies and for genome comparisons.

| **Sample ID** | **Total number of *Wolbachia* reads** | **Average read length** | **Maximum read length** | **N50** | **Average coverage** |
| --- | --- | --- | --- | --- | --- |
| *w*Cin2USA1 | 16,734 | 3,621 | 51,291 | 10,050 | 38 |
| *w*Cin2USA2 | 278,340 | 4,612 | 157,394 | 5,645 | 423 |
| *w*Cin2HUN1 | 266,306 | 4,732 | 152,050 | 5,797 | 417 |
| *w*Cin2HUN2 | 377,334 | 5,039 | 168,061 | 5,970 | 626 |
| *w*Cin2FR | 4,587 | 4,792 | 79,820 | 14,974 | 10 |
| wCin2IT | 10,076 | 3,121 | 66,580 | 9,310 | 12 |
| *w*Cer2CZ | 543,488 | 4,997 | 124,759 | 6,001 | 1673 |
| *w*Cer2HUN | 36,015 | 1,535 | 52,279 | 2,673 | 29 |
| *w*Cer2AT | 112,557 | 3,094 | 81,098 | 7,212 | 181 |
| *w*Cer2GER | 43,595 | 2,369 | 98,508 | 4,486 | 60 |

**Table S3.** Summary statistics for PacBio Sequel II and Nanopore MinION sequencing of the ten sample genomes generated and analyzed in the current study.

| **Sample** | **Host** | **Strain** | **Genome Size** | **Scaffolds** | **BUSCO Score** | **Complete BUSCOs** |
| --- | --- | --- | --- | --- | --- | --- |
| *w*Cin2USA1 | *R. cingulata* | wCin2 | 1.538351 | 1 | 99.50% | 362 |
| *w*Cin2USA2 | *R. cingulata* | wCin2 | 1.46922 | 3 | 99.50% | 362 |
| *w*Cin2HUN2 | *R. cingulata* | wCin2 | 1.531489 | 4 | 98.10% | 357 |

**Table S4.** Metrics of the genome assembly for *w*Cin2USA1, *w*Cin2USA2, and *w*Cin2HUN2. BUSCO scores were calculated using the Rickettsiales database.

| **MLST genes** | ***w*Cin2 position** | ***w*Cer2 contig:position** |
| --- | --- | --- |
| *ftsZ* | 20,655-21,851 | SOZK01000004.1:178,495-179,691 |
| *hcpA* | 939,322-940,062 | SOZK01000002.1:199,228-199,968 |
| *fbpA* | 384,743-385,639 | SOZK01000009.1:96,998-97,894 |
| *gatB* | 1,170,192-1,171,616 | SOZK01000001.1:156,281-157,705 |
| *coxA* | 1,287,090-1,288,640 | SOZK01000002.1:10,614-12,164 |
| *wsp* | 218,524-219,237 | SOZK01000008.1:64,844-65,557 |

**Table S5.** MLST gene positions in the *w*Cin2USA1 and *w*Cer2HUN2 reference genomes.

| **protein hit name** | **n hits** |
| --- | --- |
| AAA family ATPase | 7 |
| ABC transporter | 3 |
| ATP-binding protein | 2 |
| DMT family transporter | 1 |
| DNA repair | 5 |
| GPW/gp25 family protein | 2 |
| Holliday junction resolvase | 2 |
| lipase family protein | 1 |
| NTP transferase domain-containing protein | 1 |
| nucleotide sugar dehydrogenase | 1 |
| patatin-like phospholipase | 3 |
| phage | 34 |
| phospholipase D family protein | 1 |
| QueT transporter family protein | 1 |
| recombinase | 4 |
| RNA polymerase | 2 |
| S49 family peptidase | 1 |
| transcription | 5 |
| transposase | 9 |
| type II toxin-antitoxin system | 1 |
| ulp1 protease family | 1 |

**Table S6.** NCBI BLASTp microbial protein hits for the 87 of the 175 unique genes in the references *w*Cin2USA1 genome*.* The 88 genes not listed all have unknown function or are hypothetical proteins. The majority of these genes are associated with WO-phage (Bordenstein & Bordenstein, 2016; Miao, Xiao, & Huang, 2020).

**Supplement References**

Bordenstein, S. R. & Bordenstein, S. R. (2016). Eukaryotic association module in phage WO genomes from *Wolbachia*. *Nature communications*, *7*:13155. doi: 10.1038/ncomms13155

Brelsfoard, C., Tsiamis, G., Falchetto, M., Gomulski, L. M., Telleria, E., Alam, U., … Aksoy, S. (2014). Presence of Extensive *Wolbachia* Symbiont Insertions Discovered in the Genome of Its Host *Glossina morsitans morsitans*. *PLoS Neglected Tropical Diseases*, *8*(4). doi: 10.1371/journal.pntd.0002728

Conner, W. R., Blaxter, M. L., Anfora, G., Ometto, L., Rota-Stabelli, O., & Turelli, M. (2017). Genome comparisons indicate recent transfer of wRi-like *Wolbachia* between sister species *Drosophila suzukii* and *D. subpulchrella*. *Ecology and Evolution*, *7*(22), 9391–9404. doi: 10.1002/ece3.3449

Cooper, B. S., Vanderpool, D., Conner, W. R., Matute, D. R., & Turelli, M. (2019). *Wolbachia* Acquisition by *Drosophila yakuba*-Clade Hosts and Transfer of Incompatibility Loci Between Distantly Related *Wolbachia*. *Genetics*, *212*(4), 1399–1419. doi: 10.1534/genetics.119.302349

Ellegaard, K. M., Klasson, L., Näslund, K., Bourtzis, K., & Andersson, S. G. E. (2013). Comparative Genomics of *Wolbachia* and the Bacterial Species Concept. *PLOS Genetics*, *9*(4), e1003381. doi: 10.1371/journal.pgen.1003381

Gasser, M. T., Chung, M., Bromley, R. E., Nadendla, S., & Hotopp, J. C. D. (2019). Complete Genome Sequence of wAna, the *Wolbachia* Endosymbiont of *Drosophila ananassae*. *Microbiology Resource Announcements*, *8*(43). doi: 10.1128/MRA.01136-19

Gerth, M., & Bleidorn, C. (2017). Comparative genomics provides a timeframe for *Wolbachia* evolution and exposes a recent biotin synthesis operon transfer. *Nature Microbiology*, *2*(3), 16241. doi: 10.1038/nmicrobiol.2016.241

Hague, M. T. J., Caldwell, C. N., & Cooper, B. S. (2020). Pervasive Effects of *Wolbachia* on Host Temperature Preference. *MBio*, *11*(5). doi: 10.1128/mBio.01768-20

Klasson, L., Westberg, J., Sapountzis, P., Näslund, K., Lutnaes, Y., Darby, A. C., … Andersson, S. G. E. (2009). The mosaic genome structure of the *Wolbachia* wRi strain infecting *Drosophila* *simulans*. *Proceedings of the National Academy of Sciences*, *106*(14), 5725–5730. doi: 10.1073/pnas.0810753106

Lee, C.-C., Lin, C.-Y., Tseng, S.-P., Matsuura, K., & Yang, C.-C. S. (2020). Ongoing Coevolution of *Wolbachia* and a Widespread Invasive Ant, *Anoplolepis gracilipes*. *Microorganisms*, *8*(10), 1569. doi: 10.3390/microorganisms8101569

Madhav, M., Parry, R., Morgan, J. A. T., James, P., & Asgari, S. (2020). *Wolbachia* Endosymbiont of the Horn Fly (*Haematobia irritans irritans*): A Supergroup A Strain with Multiple Horizontally Acquired Cytoplasmic Incompatibility Genes. *Applied and Environmental Microbiology*, *86*(6). doi: 10.1128/AEM.02589-19

Martinez, J., Klasson, L., Welch, J. J., & Jiggins, F. M. (2021). Life and death of selfish genes: comparative genomics reveals the dynamic evolution of cytoplasmic incompatibility. *Molecular Biology and Evolution. 38*(1):2-15. doi: [doi.org/10.1093/molbev/msaa209](https://doi.org/10.1093/molbev/msaa209)

Metcalf, J. A., Jo, M., Bordenstein, S. R., Jaenike, J., & Bordenstein, S. R. (2014). Recent genome reduction of *Wolbachia* in *Drosophila recens* targets phage WO and narrows candidates for reproductive parasitism. *PeerJ*, *2*. doi: 10.7717/peerj.529

Miao, Y. H., Xiao, J. H., & Huang, D. W. (2020). Distribution and evolution of the Bacteriophage WO and Its antagonism with *Wolbachia*. *Frontiers in microbiology*, *11*, 595629. doi: 10.3389/fmicb.2020.595629

Morrow, J. L., Schneider, D. I., Klasson, L., Janitz, C., Miller, W. J., & Riegler, M. (2020). Parallel Sequencing of *Wolbachia* wCer2 from Donor and Novel Hosts Reveals Multiple Incompatibility Factors and Genome Stability after Host Transfers. *Genome Biology and Evolution*, *12*(5), 720–735. doi: 10.1093/gbe/evaa050

Saha, S., Hunter, W. B., Reese, J., Morgan, J. K., Marutani-Hert, M., Huang, H., & Lindeberg, M. (2012). Survey of Endosymbionts in the *Diaphorina citri* Metagenome and Assembly of a *Wolbachia* wDi Draft Genome. *PLoS ONE*, *7*(11). doi: 10.1371/journal.pone.0050067

Salzberg, S. L., Puiu, D., Sommer, D. D., Nene, V., & Lee, N. H. (2009). Genome sequence of the *Wolbachia* endosymbiont of *Culex quinquefasciatus* JHB. *Journal of Bacteriology*, *191*(5), 1725. doi: 10.1128/JB.01731-08

Sinha, A., Li, Z., Sun, L., & Carlow, C. K. S. (2019). Complete Genome Sequence of the *Wolbachia* wAlbB Endosymbiont of *Aedes albopictus*. *Genome Biology and Evolution*, *11*(3), 706–720. doi: 10.1093/gbe/evz025

Sutton, E. R., Harris, S. R., Parkhill, J., & Sinkins, S. P. (2014). Comparative genome analysis of *Wolbachia* strain wAu. *BMC Genomics*, *15*(1), 928. doi: 10.1186/1471-2164-15-928

Wallau, G. L., da Rosa, M. T., De Ré, F. C., & Loreto, E. L. S. (2016). *Wolbachia* from *Drosophila incompta*: Just a hitchhiker shared by Drosophila in the New and Old World? *Insect Molecular Biology*, *25*(4), 487–499. doi: 10.1111/imb.12237

Wang, X., Xiong, X., Cao, W., Zhang, C., Werren, J. H., & Wang, X. (2019). Genome Assembly of the A-Group *Wolbachia* in *Nasonia oneida* Using Linked-Reads Technology. *Genome Biology and Evolution*, *11*(10), 3008–3013. doi: 10.1093/gbe/evz223

Woolfit, M., Iturbe-Ormaetxe, I., Brownlie, J. C., Walker, T., Riegler, M., Seleznev, A., … O’Neill, S. L. (2013). Genomic Evolution of the Pathogenic *Wolbachia* Strain, wMelPop. *Genome Biology and Evolution*, *5*(11), 2189–2204. doi: 10.1093/gbe/evt169

Wu, M., Sun, L. V., Vamathevan, J., Riegler, M., Deboy, R., Brownlie, J. C., … Eisen, J. A. (2004). Phylogenomics of the Reproductive Parasite Wolbachia pipientis wMel: A Streamlined Genome Overrun by Mobile Genetic Elements. *PLOS Biology*, *2*(3), e69. Doi: 10.1371/journal.pbio.0020069
